# Comparative predictive power of serum vs plasma proteomic signatures in feto-maternal medicine

**DOI:** 10.1016/j.xagr.2023.100244

**Published:** 2023-06-12

**Authors:** Camilo Espinosa, Said Mohammed Ali, Waqasuddin Khan, Rasheda Khanam, Jesmin Pervin, Joan T. Price, Sayedur Rahman, Tarik Hasan, Salahuddin Ahmed, Rubhana Raqib, Monjur Rahman, Shaki Aktar, Muhammad I. Nisar, Javairia Khalid, Usha Dhingra, Arup Dutta, Saikat Deb, Jeffrey S.A. Stringer, Ronald J. Wong, Gary M. Shaw, David K. Stevenson, Gary L. Darmstadt, Brice Gaudilliere, Abdullah H. Baqui, Fyezah Jehan, Anisur Rahman, Sunil Sazawal, Bellington Vwalika, Nima Aghaeepour, Martin S. Angst

**Affiliations:** 1 Department of Anesthesiology, Perioperative and Pain Medicine, Stanford University School of Medicine, Stanford, CA (Mr Espinosa and Drs Gaudilliere, Aghaeepour and Angst); 2 Public Health Laboratory Ivo de Carneri, Zanzibar, Pemba, Tanzania (Messrs Ali, Dutta, and Deb); 3 Biorepository and Omics Research Group, Department of Pediatrics and Child Health, Faculty of Health Sciences, Medical College, Aga Khan University, Karachi, Pakistan (Drs Khan and Nisar, Ms Khalid, and Dr Jehan); 4 Newborn Health, Department of International Health, Johns Hopkins Bloomberg School of Public Health, Baltimore, MD (Drs Khanam and Baqui); 5 Maternal and Child Health Division, International Centre for Diarrhoeal Disease Research, Dhaka, Bangladesh (Mr Pervin, Mr M. Rahman, and Drs Aktar and A. Rahman); 6 Department of Obstetrics and Gynecology, The University of North Carolina at Chapel Hill, Chapel Hill, NC (Drs Price and Stringer); 7 Projahnmo Research Foundation, Dhaka, Bangladesh (Dr Rahman, Mr Hasan, and Dr Ahmed); 8 Infectious Diseases Division, International Centre for Diarrhoeal Disease Research, Dhaka, Bangladesh (Dr Raqib); 9 Johns Hopkins Bloomberg School of Public Health, Johns Hopkins University, Baltimore, MD (Ms Dhingra and Dr Sazawal); 10 Center for Public Health Kinetics, New Delhi, India (Ms Dhingra, Messrs Dutta and Drs Deb, and Sazawal); 11 Department of Pediatrics, Stanford University School of Medicine, Stanford, CA (Drs Wong, Shaw, Stevenson, Darmstadt, Gaudilliere and Aghaeepour); 12 Department of Obstetrics and Gynecology, UNC School of Medicine, University of Zambia, Lusaka, Zambia (Dr Vwalika); 13 Department of Biomedical Informatics, Stanford University School of Medicine, Stanford, CA (Dr Aghaeepour).

**Keywords:** biobanking, biomarker, biorepository, cohort study, gestational age, maternal health, multivariate model, plasma, prediction, pregnancy, proteins, proteomics, serum

## Abstract

**BACKGROUND:**

Blood proteins are frequently measured in serum or plasma, because they provide a wealth of information. Differences in the ex vivo processing of serum and plasma raise concerns that proteomic health and disease signatures derived from serum or plasma differ in content and quality. However, little is known about their respective power to predict feto-maternal health outcomes. Predictive power is a sentinel characteristic to determine the clinical use of biosignatures.

**OBJECTIVE:**

This study aimed to compare the power of serum and plasma proteomic signatures to predict a physiological pregnancy outcome.

**STUDY DESIGN:**

Paired serum and plasma samples from 73 women were obtained from biorepositories of a multinational prospective cohort study on pregnancy outcomes. Gestational age at the time of sampling was the predicted outcome, because the proteomic signatures have been validated for such a prediction. Multivariate and cross-validated models were independently derived for serum and plasma proteins.

**RESULTS:**

A total of 1116 proteins were measured in 88 paired samples from 73 women with a highly multiplexed platform using proximity extension technology (Olink Proteomics Inc, Watertown, MA). The plasma proteomic signature showed a higher predictive power (R=0.64; confidence interval, 0.42–0.79; *P*=3.5×10^-6^) than the serum signature (R=0.45; confidence interval, 0.18–0.66; *P*=2.2×10^-3^). The serum signature was validated in plasma with a similar predictive power (R=0.58; confidence interval, 0.34–0.75; *P*=4.8×10^-5^), whereas the plasma signature was validated in serum with reduced predictive power (R=0.53; confidence interval, 0.27–0.72; *P*=2.6×10^-4^). Signature proteins largely overlapped in the serum and plasma, but the strength of association with gestational age was weaker for serum proteins.

**CONCLUSION:**

Findings suggest that serum proteomics are less informative than plasma proteomics. They are compatible with the view that the partial ex-vivo degradation and modification of serum proteins during sample processing are an underlying reason. The rationale for collecting and analyzing serum and plasma samples should be carefully considered when deriving proteomic biosignatures to ascertain that specimens of the highest scientific and clinical yield are processed. Findings suggest that plasma is the preferred matrix.

**AJOG Global Reports at a Glance**

**Why was this study conducted?**

This study aimed to address the unanswered question of whether it is important to collect and analyze serum or plasma samples when deriving proteomic signatures to predict a feto-maternal health outcome.

**Key findings**

The plasma proteomic signature possessed markedly stronger power to predict the physiological health outcome of gestational age when compared with the serum proteomic signature.

**What does this add to what is known?**

The novelty of this study is the direct comparison of the predictive power of serum and plasma proteomic signatures, a sentinel metric to determine the clinical use of biosignatures. Findings suggest that plasma is the preferred matrix.

## Introduction

Among the multiple biologic layers explored in omic efforts, proteins provide a wealth of information on human health and disease.^1^^,^^2^ Proteins are critical effector molecules that respond to environmental factors and mediate gene functions. The comprehensive characterization of the blood proteome offers insight into different organ systems and their coordination of human health. The composition of the blood proteome and its expression patterns has enormous potential for the personalized monitoring of feto-maternal health, risk prediction, accurate disease diagnosis, and therapeutic surveillance.^3^, ^4^, ^5^, ^6^

Major matrices for interrogating the blood proteome include serum and plasma. However, these matrices differ in terms of their ex vivo processing. Spontaneous clot formation at room temperature is part of the typical protocol to separate serum, whereas clot formation is actively prevented when separating plasma. Clot formation is associated with the degradation and modification of serum proteins and the release of proteins from platelets, leukocytes, and the clot itself.^7^, ^8^, ^9^ Thus, proteomic health and disease signatures in serum and plasma may differ in content and quality, a proposition that remains the subject of significant debate.

Although it is well established that associations between proteins measured in serum and plasma are imperfect, little is known about the functional consequences of such divergence when deriving proteomic signatures as predictive, prognostic, or therapeutic biomarkers.^7^^,^^8^ A sentinel characteristic of biomarkers when determining their clinical use is their predictive power, which relates to their positive and negative predictive values. In this study, we addressed the critical question of whether proteomic signatures derived from either serum or plasma differ in their predictive power. For this study, a physiological clinical outcome of pregnancy, namely gestational age at the time of blood sampling, was selected as the predicted outcome, because proteomic signatures have recently been validated for such prediction.^10^^,^^11^

## Material and Methods

### Study design and participants

Blood samples for this analysis were obtained from biorepositories of 2 multinational prospective cohort studies of maternal and fetal outcomes. The Alliance for Maternal and Newborn Health Improvement (AMANHI) cohorts were enrolled in Bangladesh (Sylhet), Pakistan (Karachi), and Tanzania (Pemba Island), and the Global Alliance to Prevent Prematurity and Stillbirth (GAPPS) cohorts were enrolled in Bangladesh (Matlab) and Zambia (Lusaka).^12^^,^^13^ The AMANHI and GAPPS protocols were very similar with respect to visit schedule and specimen collection procedures. The current combined cohort was produced through an exhaustive harmonization effort conducted by the study teams from all sites. All participating women provided written informed consent for the original studies and for future use of specimens. The institutional review board (IRB) of Stanford University approved this study, and the IRBs of the respective countries allow for the extended analyses. Specimen transfer was regulated by necessary material transfer and data-use agreements. Study participants were identified by participating sites either through community surveillance by community health workers or through recruitment at antenatal facilities. For this report, we followed the Strengthening the Reporting of Observational Studies in Epidemiology guidelines.^14^

The inclusion criteria were enrollment during early pregnancy (8–19 gestational weeks [GWs]), positive urine pregnancy test, and gestational age confirmed by ultrasound. Harmonized protocols were used by trained study sonographers to derive gestational age with accurate biometric parameters, namely crown-rump length for fetuses <14 GWs and biparietal diameter and femur length for fetuses ≥14 GWs.^15^^,^^16^ Quality assurance measures included an expert review of sonographic images within sites for a random sample of at least 10% of the study subjects and by an external consultant in obstetrics and gynecology for a random sample of at least 5% of subjects. Trained study field workers collected epidemiologic and phenotypical data after the initial visit (GWs 9–19), at GWs 24 to 28 or GWs 32 to 36, after GW 37, and postpartum (days 0–6 and 42–60) either through household visits or telephonic conversations. Our study cohort consisted of 73 pregnant women from Sylhet (n=17), Karachi (n=15), Pemba Island (n=16), Matlab (n=15), and Lusaka (n=10).

### Collection and analysis of biologic specimens

Blood was collected by trained phlebotomists, and the serum and plasma samples were isolated and aliquoted using uniform protocols. Samples analyzed in this study were collected during the initial visit (GW 8–19) and at a subsequent visit (GWs 24–28). Blood collected in ethylendiaminetetraacetic acid tubes (plasma) were kept on ice until centrifuged at 4°C and 2000 rpm for 15 minutes within a target interval of 30 minutes between collection and processing. Vials containing blood collected for serum retrieval were set upright in a rack at room temperature for 30 minutes to allow for clot formation. Plasma and serum were then separated, aliquoted, and stored at −80°C until shipment as a single batch on dry ice and under continuous temperature monitoring.

Study sites contributed a total of 88 paired and simultaneously collected plasma and serum samples. A total of 57 women contributed 1 paired sample collected during the first or second study visit, and 16 women contributed 2 paired samples collected at subsequent study visits.

Plasma and serum samples were analyzed with a highly multiplexed platform using proximity extension technology to quantify 1161 proteins (Olink Proteomics Inc, Watertown, MA).^17^^,^^18^ For this study, 13 panels were used, each measuring 92 different proteins in 1 μL of plasma or serum. Briefly, each protein was detected by a matched pair of antibodies coupled to unique and partially complementary oligonucleotides. When in close proximity, a new and protein-specific DNA reporter sequence forms by hybridization and extension, which is then amplified and quantified by real-time polymerase chain reaction. Relative amounts of protein were quantified as normalized protein expression (NPX) in which case an increase of 1 NPX corresponded to a doubling of the relative protein concentration. Technical details regarding the derivation of NPX and the metrics used to ascertain assay performance and sample quality are available from the provider (Olink Proteomics Inc).^19^

### Computational analysis

Spearman's rank correlation coefficients were calculated to assess the strength of the association between (1) the same proteins measured in serum and plasma and (2) the same proteins either measured in serum or plasma and gestational age at the time of sampling.

Multivariate proteomic models predicting gestational age at the time of sampling were independently derived for serum and plasma proteins using 4 separate machine learning approaches. Regularized linear regression with elastic net and gradient-boosted trees (XGBoost) were evaluated using 2 cross-validation (CV) strategies, namely a 5-fold CV or a 2-layer repeated CV scheme to prevent overfitting and to derive model performance estimates. The XGBoost model with the 2-layer repeated CV scheme performed best in predicting gestational age for each matrix (serum or plasma) and when tested across matrices. This model and CV strategy were chosen for the final analysis. In brief, patients were randomly split into a training set (80%) and a test set (20%). The XGBoost model was trained on the training set and then used to predict the gestational age at the time of sampling for patients in the test set. This procedure was performed 100 times using different training and test sets in each iteration, and final test predictions for each patient were generated by averaging the patient predictions from the iterations in which the patient was in the test set. Model performance was assessed using the Pearson correlation coefficient (Pearson R).

To compare the predictive model derived in plasma with the predictive model derived in serum, the same train-test splits were used for both modalities in each iteration of the CV procedure and when assessing the performance of each model when tested in the other matrix.

## Results

### Study participants

The median maternal age was 26 years (IQR, 21–30), the median body mass index was 23.6 kg/m^2^ (IQR, 20.9–26.9), the median parity was 1 (IQR, 0–3), and the median gravidity was 3 (IQR, 2–5). Pregnancy-induced hypertension was reported in 2 women, whereas diabetes mellitus was not observed. The median gestational age at birth was 37.6 weeks (IQR, 33.7–39.6). A total of 37 women delivered at term at a median gestational age of 39.6 weeks (IQR, 39.3–40.0), and 36 women delivered preterm at a median gestational age of 33.7 weeks (IQR, 31.5–35.3).

### Plasma and serum samples

A total of 43 paired samples were collected during the first study visit at a median gestational age of 12.1 weeks (IQR, 10.6–16.1), and 45 paired samples were collected during the second study visit at a median gestational age of 23.7 weeks (IQR, 22.2–24.3). Gestational age at sampling did not differ between study sites.

### Assay and sample quality metrics

All plates passed quality control, as did 98.2% of the plasma and 98.5% of the serum samples. Of all the assayed proteins, 84.4% were detected in more than 75% of the plasma samples, whereas 85.5% were detected in more than 75% of the serum samples. The median intra-assay coefficient of variation was 6% (range, 4–8) and the median inter-assay coefficient of variation was 11% (range, 9–13) for plasma samples. The respective values were 6% (range, 4–8) and 9% (range, 6–11) for serum samples. These quality metrics met expectations and are comparable with those previously reported in similar cohorts.^18^

### Correlations

**Figure 1:**
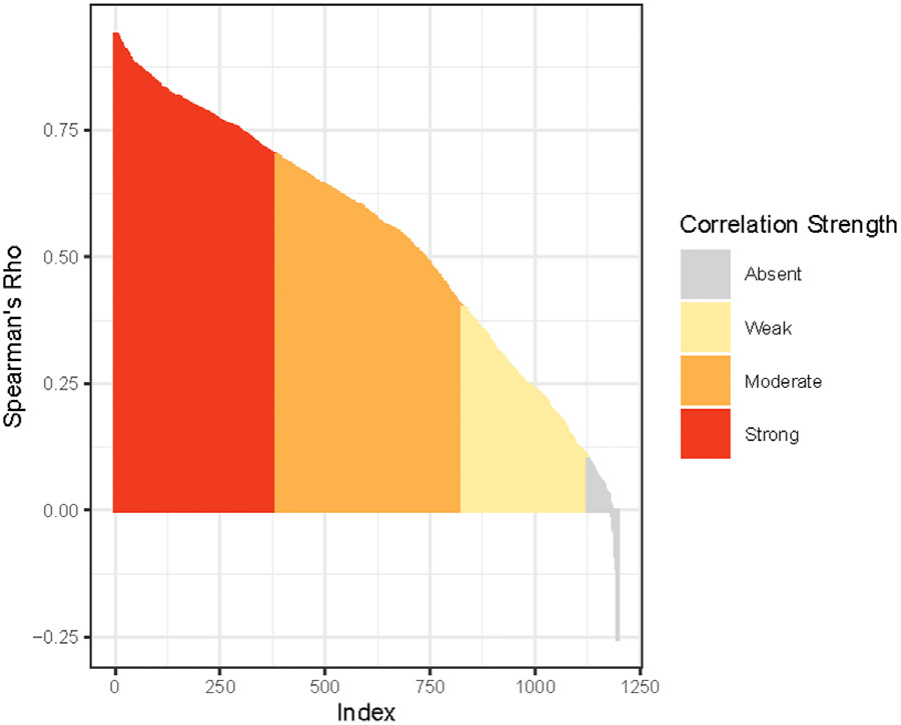
Association strength between serum and plasma proteins The Spearman correlation coefficients for 1161 proteins measured simultaneously in serum and plasma varied widely. Proteins are ranked by Spearman rho from highest to lowest and color codes indicate strong, moderate, weak, and absent associations.

The correlation coefficients for individual proteins measured in serum and plasma varied widely (Figure 1). Associations were strong (R≥0.70) for 28%, moderate (R, 0.40–0.69) for 40%, weak (R, 0.10–0.39) for 25%, and absent (R≤0.10) for 7% of proteins. Associations were generally conserved when calculated separately for the AMANHI and the GAPPS cohorts with a median absolute difference in Spearman's Rho between cohorts of 0.13 and comparable correlation strengths for most proteins.

Espinosa. Serum vs plasma proteomic signatures. Am J Obstet Gynecol Glob Rep 2023.

### Predictive models

**Figure 2:**
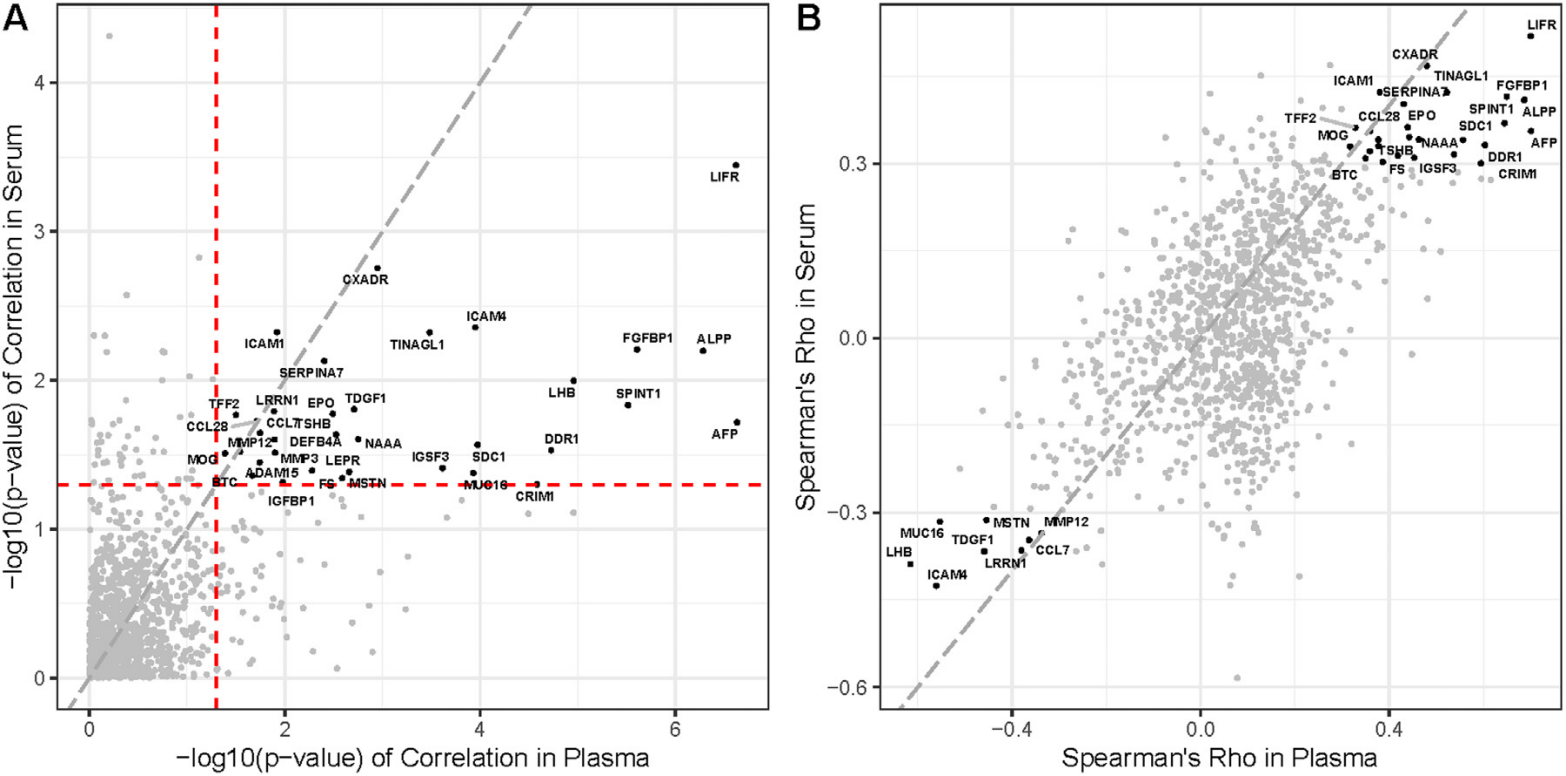
Comparative predictive power of model proteins **A,** Proteins in serum and plasma significantly associated with gestational age are labeled by their gene names. The *red dashed lines* represent a *P* value of.05, and the *gray dashed line* is the line of identity (y=x). Proteins generally possessed higher predictive power when measured in plasma. **B,** Shown are the correlation coefficients for serum and plasma proteins significantly associated with gestational age. The *gray dashed line* is the line of identity. The majority of correlation coefficients indicate stronger associations for plasma than for serum proteins.

Cross-validated multivariate models predicting gestational age at the time of sampling were derived independently for serum and plasma using the first collection time points. The plasma model possessed higher predictive power (Pearson's R=0.64; 95% CI, 0.42–0.79; *P*=3.5 × 10^−6^; root-mean-square error [RMSE] 2.7 weeks) than the serum model (R=0.45; 95% CI, 0.18–0.66; *P*=2.2 × 10^−3^; RMSE 3.1 weeks). The model derived in plasma was validated in serum with reduced predictive power (R=0.53; 95% CI, 0.27–0.72; *P*=2.6 × 10^−4^; RMSE 2.9 weeks), whereas the model derived in serum was validated in plasma with similar predictive power (R=0.58; 95% CI, 0.34–0.75; *P*=4.8 × 10^−5^; RMSE 3.0 weeks). The proteins in the serum and plasma that were significantly associated with gestational age at sampling were largely overlapping (Figure 2), but most of these proteins showed a weaker association when measured in serum than when measured in plasma.

Espinosa. Serum vs plasma proteomic signatures. Am J Obstet Gynecol Glob Rep 2023.

## Discussion

### Principal findings

The plasma proteomic signature was more powerful than the serum proteomic signature for predicting a physiological pregnancy outcome, namely gestational age at the time of sampling. The proteins included in the serum and plasma signatures were similar. However, the association between individual proteins included in the signatures were consistently weaker for the serum signature. This indicates that predictive information contained in serum is preserved in plasma, whereas some predictive information contained in plasma is not preserved in serum. A significant concern regarding serum proteins is their partial degradation and modification by proteolysis during ex vivo processing. The proteomic serum signature reported here looked similar but was weaker when compared with the plasma proteomic signature, which is compatible with the partial ex vivo degradation and modification of serum proteins.

### Results

Although the results regarding the predictive power of serum and plasma signatures are novel, previous work echoes the finding of a wide-ranging strength of associations when comparing serum and plasma levels of an array of proteins.^7^^,^^8^ In this study, correlations were strong for about 30% of the 1161 measured proteins. By comparison, a recent study assaying 358 proteins from 22 subjects with the same platform under well-controlled conditions reported strong correlations for about 55% of the measured proteins.^8^ The discrepancy may reflect unique challenges faced by large and difficult to conduct cohort studies, including the strict adherence to ideal sampling and processing protocols. As such, current results may provide reasonable estimates for such efforts.

### Clinical implications

The reported results have important implications when considering investments in precision medicine and biobanking efforts including proteomics. Establishing biobanks is expensive, and sustaining biorepositories in the longterm is a logistical and financial challenge. As an example, the US Congress has allocated $1.02 billion for the “All of US” research program since 2015 and has authorized an additional $1.14 billion through 2026 via the 21st Century Cures Act.^20^ Similarly, current per sample costs for highly multiplexed proteomic analyses are several hundred dollars.^21^^,^^22^ Consequently, biospecimens collected, made available, and analyzed through biobanking efforts should be of the highest possible scientific yield.

### Research implications

From a conceptional perspective, plasma proteomics may reflect in vivo physiology and pathophysiology more closely than serum proteomics, because serum samples are subjected to ex vivo processing steps that can alter their qualitative and quantitative content. However, the generalizability of our results will require further comparative studies for different health outcomes and healthcare settings. For example, serum and plasma samples may be equivalent when measuring proteins that are not susceptible to ex vivo degradation, including immunoglobulins.^23^ Based on our findings, future studies should extend beyond correlational analysis, which has commonly been used when comparing serum and plasma samples. They should include metrics relevant for determining the clinical use of a particular biosignature such as its predictive power.

### Strengths and limitations

This study provided a direct comparison of the relative power of serum and plasma proteomic signatures for predicting a relevant health outcome. Predictive power is a sentinel characteristic that can be used to determine the clinical use of biosignatures as it relates to their positive and negative predictive values.

The study does not address whether results are generalizable if different proteomic assays are used. In this context, a previous study of 17 pregnant women is noteworthy. Although the comparison of serum and plasma was not the primary objective, a subset of data suggested that proteomic signatures predicting gestational age were more powerful when derived from plasma rather than from serum.^24^ Proteins were measured with a 62-plex assay from eBioscience, suggesting consistency of findings when using 2 different assays.

Protein levels were determined with an assay using oligonucleotide-linked antibodies. Concerns have been raised that interference with certain biologic compounds, particularly autoantibodies, may be specific to assays using oligonucleotides.^25^ Given the potential of technology-dependent limitations, results validated with different assay technologies are generally more robust.

## Conclusion

Findings suggest that serum proteomics are less informative than plasma proteomics. The rationale for collecting and analyzing serum and plasma samples should be carefully considered when deriving proteomic biosignatures to ascertain that specimens of the highest scientific and clinical yield are processed. Findings suggest that plasma is the preferred matrix.
